# Reactivity of Coproheme Decarboxylase with Monovinyl, Monopropionate Deuteroheme

**DOI:** 10.3390/biom13060946

**Published:** 2023-06-06

**Authors:** Gaurav Patil, Hanna Michlits, Paul G. Furtmüller, Stefan Hofbauer

**Affiliations:** Department of Chemistry, Institute of Biochemistry, University of Natural Resources and Life Sciences, Muthgasse 18, A-1190 Vienna, Austria

**Keywords:** coproheme decarboxylase, stopped-flow spectroscopy, HPLC, mass spectrometry

## Abstract

Coproheme decarboxylases (ChdCs) are terminal enzymes of the coproporphyrin-dependent heme biosynthetic pathway. In this reaction, two propionate groups are cleaved from the redox-active iron-containing substrate, coproheme, to form vinyl groups of the heme *b* product. The two decarboxylation reactions proceed sequentially, and a redox-active three-propionate porphyrin, called monovinyl, monopropionate deuteroheme (MMD), is transiently formed as an intermediate. While the reaction mechanism for the first part of the redox reaction, which is initiated by hydrogen peroxide, has been elucidated in some detail, the second part of this reaction, starting from MMD, has not been studied. Here, we report the optimization of enzymatic MMD production by ChdC and purification by reversed-phase chromatography. With the obtained MMD, we were able to study the second part of heme *b* formation by actinobacterial ChdC from *Corynebacterium diphtheriae*, starting with Compound I formation upon the addition of hydrogen peroxide. The results indicate that the second part of the decarboxylation reaction is analogous to the first part, although somewhat slower, which is explained by differences in the active site architecture and its H-bonding network. The results are discussed in terms of known kinetic and structural data and help to fill some mechanistic gaps in the overall reaction catalyzed by ChdCs.

## 1. Introduction

**Figure 1:**
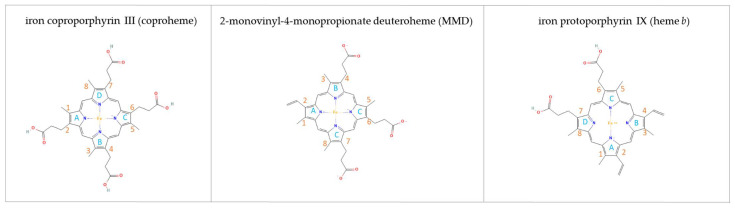
Overview of relevant porphyrins involved in the reaction of coproheme decarboxylase. Representations of all porphyrins were obtained from the PubChem databank (https://pubchem.ncbi.nlm.nih.gov, accessed on 22 May 2023). ChdCs use iron coproporphyrin III (coproheme) as a substrate (PubChem-ID: 70678602). The three-propionate intermediate, called 2-monovinyl-4- monopropionyl deuteroheme (MMD), is the transiently formed intermediate and is found under the name “harderoheme” in the PubChem databank (PubChem-ID 134820392). Iron protoporphyrin IX (heme *b*, protoheme) is the final product of the reaction catalyzed by coproheme decarboxylases (PubChem-ID: 444097). Pyrrole rings are labeled in cyan and porphyrin substituents numbering is shown in orange.

The reaction mechanism of coproheme decarboxylases (ChdCs) has been under investigation for some time and has already led to a thorough understanding of many aspects underlying this catalytic process. This redox active enzyme is found in the so-called “coproporphyrin-dependent” heme biosynthesis pathway, which is mainly utilized by monoderm bacteria [1,2,3,4,5]. Within this pathway, ChdCs are responsible for the final step—that is, the oxidative decarboxylation of two propionate side chains of the substrate coproheme, releasing two carbon dioxide molecules to yield heme *b* (Figure 1), which has two vinyl groups at the respective positions [6]. Two equivalents of hydrogen peroxide are necessary to perform both decarboxylation reactions [7]. The hydrogen peroxide is deprotonated by a distal base (H118) in actinobacterial ChdCs. In ChdCs, the substrate coproheme (iron coproporphyrin III, Figure 1) acts also as the redox cofactor, as the ferric iron is oxidized by deprotonated hydrogen peroxide yielding Compound I (Fe(IV)... porph^●^) [8,9]. Consecutively, Compound I is reduced to Compound I* (Fe(IV)... aa^●^), forming a catalytically essential tyrosyl radical (Y135) [8,9,10], which then facilitates the nucleophilic attack on the β-carbon of the propionate side chain to release carbon dioxide, forming the vinyl group and reducing Compound II to the ferric form of the three-propionate intermediate (2-monovinyl, 4-monopropionate, deuteroheme, MMD (Figure 1)). This tyrosyl radical has been shown to be essential for both decarboxylation reactions [11]. Therefore, a reorientation of the three-propionate intermediate was proposed and proven spectroscopically by resonance Raman studies [8], as well as structurally by X-ray crystallographic studies [9]. The mechanism of reorientation—rotation in the active site versus release and rebinding—is not easy to identify, but spectroscopic in-solution studies, as well as computational studies, are in agreement with a rotation of MMD within the active site, suggesting a gating-like function by two active-site tryptophan residues [12,13].

**Figure 2:**
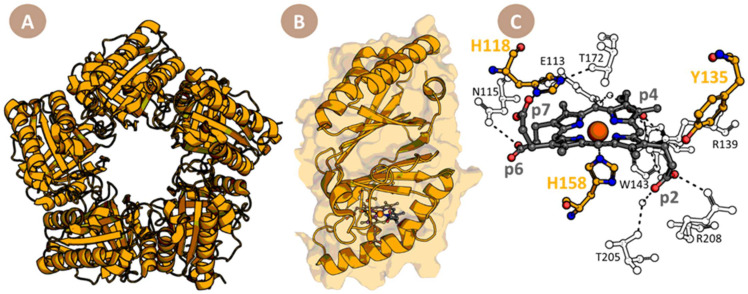
Overall structure of *Cd*ChdC. (**A**) Cartoon representation of pentameric *Cd*ChdC (pdb-code: 6XUC; yellow). (**B**) Subunit structure (yellow cartoon and semi-transparent surface) of coproheme–*Cd*ChdC. (**C**) Active site of coproheme–*Cd*ChdC; catalytically important active site residues are displayed as a yellow ball and stick model (carbon atoms) and coproheme as a grey ball and stick model. All oxygen atoms are presented in red, nitrogen atoms in dark blue, and dashed lines for H-bonding interactions are shown in grey. Ball and stick models of residues involved in H-bonding interactions with coproheme propionate groups and catalytic residues are shown as contours.

ChdCs are pentameric enzymes (Figure 2A), and each subunit is built by two ferrodoxin-like folds connected by a loop (Figure 2B). The active site of ChdCs is built to stabilize the substrate via H-bonding interactions, especially interacting with propionate 2 (p2) and propionate 4 (p4), which are the ones to be cleaved off [14,15,16]. Coproheme iron is coordinated by a proximal histidine (H158), which is not involved in any hydrogen bonding network [14,17,18,19] (Figure 2C). This lack of H-bonding on the proximal side not only modulates the redox potential of the Fe(III)/Fe(II) couple [20,21], but also has two other major implications: (i) the final substrate, heme *b*, is not tightly bound to ChdC and can be easily delivered to heme-accepting apo-proteins [22,23]; and (ii) it is a necessary precondition for the proposed reorientation of MMD after the first decarboxylation in the active site, giving the appropriate amount of freedom to enable reorientation [12,13].

Generally, ChdCs from different phylogenetic lineages (actinobacteria or firmicutes, respectively) are proposed to employ the same reaction mechanism, although specific details do vary between different classes of ChdCs. Activation of hydrogen peroxide by deprotonation is efficiently performed only in actinobacterial ChdC (e.g., from *Corynebacterium diphtheriae*), in which a distal histidine residue has been identified that enhances the Compound I formation rate [9]. This distal histidine is located on a flexible loop linking the N-terminal and the C-terminal ferredoxin-like fold domain of ChdCs, and is not present in firmicute ChdCs (e.g., *Listeria monocytogenes*) [24]. In *Cd*ChdC, mutation of this distal histidine residue to a phenylalanine (H118F) leads to accumulation of MMD due to hindrance of the in situ reorientation [13]. Further differences are identified in the active site between actinobacterial and firmicute ChdCs, which, for instance, affect the H-bonding network of p4 and the tryptophan pair proposed to act as a supporting gate in MMD rotation [12,25].

In this work we investigate the reaction kinetics of the second decarboxylation step, starting from MMD, which has not been studied so far. MMD cannot be purchased from a commercial partner (in contrast to coproheme) but has to be synthesized, which is an expensive and demanding task [6]. We have established an enzymatic production protocol for MMD, utilizing *Cd*ChdC wild-type or the H118F variant, and further optimized MMD purification via a C18 column, performed on an HPLC system. With the obtained MMD, we were able to study pre-steady-state kinetics and prove that the Compound I formation of the three-propionate porphyrin intermediate upon reaction with hydrogen peroxide is the initial reaction for the second decarboxylation step, yielding the final product, heme *b.* The reaction of coproheme-*Cd*ChdC wild-type with the oxidant, hydrogen peroxide, followed by UV–Vis stopped-flow spectroscopy led to a complex multiphasic reaction, as we have previously published [9]. Here, we are able to further dissect this multiphasic reaction and provide evidence for the important step of Compound I formation of the three-propionate intermediate MMD. The results are discussed with respect to the first part of ChdC’s decarboxylation reaction and with the known structure–function relationships.

## 2. Materials and Methods

### 2.1. Expression and Purification of CdChdC Wild-Type and Variants

*Cd*ChdC wild-type and variants were expressed and purified as described previously [9]. *Cd*ChdC Y135A and H118F mutants were produced by site-directed mutagenesis, using the *Cd*ChdC wild-type plasmid as the template, and expressed and purified. Site-directed mutagenesis was performed using the QuikChange Lightning kit [9,13].

### 2.2. Coproheme Decarboxylase Activity

The *Cd*ChdC wild-type and Y135A mutant activity was calculated using the UV–Vis electronic absorption spectra recorded by means of a Cary 60 spectrophotometer (Agilent Technologies, Santa Clara, CA, USA) with a scan rate of 600 nm min^−1^ and a resolution of 1.5 nm. To analyze their respective activities, 18 μM of recombinant enzyme was added to 9 μM of coproheme in 1000 μL of 100 mM phosphate buffer solution, pH 7, to form *Cd*ChdC–coproheme complexes. The *Cd*ChdC–coproheme complexes were eventually titrated to heme *b* complexes by adding small aliquots of a 1 mM H_2_O_2_ stock solution. The complete heme *b* formation was followed by the recording of the electronic absorption spectra of the wild-type and variant upon the addition of three equivalents (eqs.) of H_2_O_2_.

Samples from this solution (10 μL) were drawn and analyzed using a Dionex Ultimate 3000 system directly linked to a QTOF mass spectrometer (maXis 4G ETD, Bruker), which was equipped with the standard ESI source in the positive ion mode using an optimized protocol to simultaneously detect small masses of porphyrins and the entire protein [7].

### 2.3. Production and Purification of Reaction Intermediate MMD

Initially, the *Cd*ChdC H118F variant was used to set up and optimize the protocol for the enzymatic production and purification of MMD due to its inability to form heme *b*. Nevertheless, in the end, the optimized purification protocol, with the final chromatography settings (solvents, gradient, column), was perfectly able to baseline separate coproheme, MMD, and heme *b*. Therefore, *Cd*ChdC wild-type was used to produce the MMD, which was obtained for measurement, as the oxidant-excess needed to obtain the maximal amount of MMD was much lower than for the *Cd*ChdC H118F variant.

The wild-type *Cd*ChdC coproheme complex was prepared by adding 8 μM coproheme solution to excess of *Cd*ChdC apoprotein (16 μM) diluted in 100 mM phosphate buffer to a final reaction volume of 10 mL. The reconstituted protein was stirred continuously at 25 °C. The addition of 2 equivalents of H_2_O_2_ induced the conversion of coproheme to MMD and then to heme *b*. The reaction was stopped by addition of cyanide and the newly formed complex of *Cd*ChdC MMD was obtained by a modified extraction method by Teale [23,26]. In detail, HCl was added on ice to the reacted porphyrin-containing *Cd*ChdC until its pH 2 was reached. An equal amount of butanol was used as an organic solvent to separate the porphyrins from protein in the reaction mixture. Additional rounds of butanol addition can be used to separate the remaining porphyrins.

The mixture containing porphyrins (coproheme, MMD, and heme *b*) was applied on to the C18 column (Phenomenex Jupiter C18 5u 300A) using an HPLC system (Shimadzu prominence LC20) equipped with a refractive index detector (RID-10A, Shimadzu), a diode array detector (SPD-M20A, Shimadzu), and a fraction collector. A gradient from 35% Solvent B and 65% Solvent A (Solvent A: 80 mM ammonium formeate and 10 % acetonitrile; Solvent B: methanol and 10% acetonitrile) to 95% Solvent B for 16 min at 45 °C was applied. The flow rate was set to 0.9 mL min^−1^. The solvent containing the MMD fraction was further lyophilized and dissolved in phosphate buffer pH 7. Relative amounts of the formed MMD, heme *b*, as well as oxidized coproheme upon the addition of hydrogen peroxide, were determined using mass spectrometry, identically as described above in the description of coproheme decarboxylase activity.

### 2.4. Stopped-Flow Spectroscopy

Pre-steady-state spectroscopic changes were measured with a stopped-flow apparatus (SX-18MV equipped with diode array detector) from Applied Photophysics. All measurements were performed in triplicates at 25 °C. Typically, 4 μM *Cd*ChdC (in 100 mM phosphate buffer, pH 7.0) were added to 2 µM MMD in order to ensure complete binding of the three-propionate intermediate, directly before the measurements. H_2_O_2_ concentration varied from 0.02 to 0.14 mM.

To determine the rate constants for hydrogen peroxide-mediated Compound I formation, time traces were taken at 393 nm. *K*_obs_ values for each concentration were determined by fitting the respective time traces with a single-exponential fit. *K*_obs_ values were fitted using the Pro-Data viewer software and plotted against the respective H_2_O_2_ concentration. From the slope of the plot, the *k*_app_ of Compound I formation can be obtained [9,27,28,29].

## 3. Results

### 3.1. MMD Production and Purification

**Figure 3:**
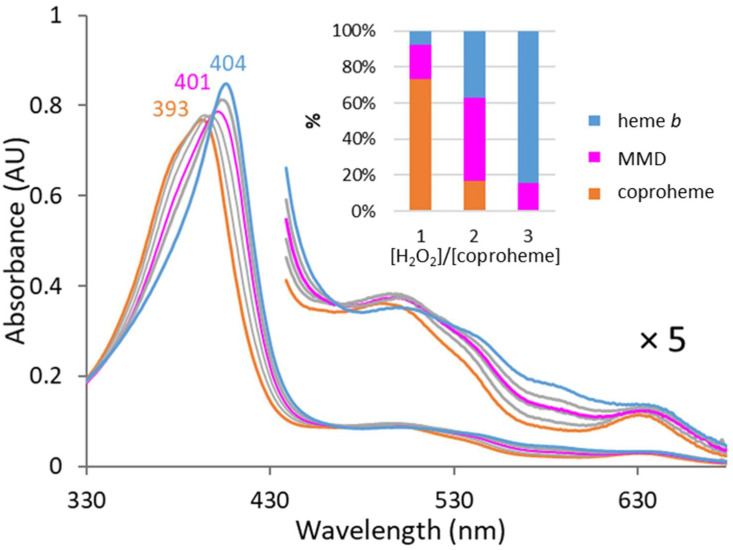
Estimation of optimal oxidant concentration. Activity of *Cd*ChdC wild-type was monitored according as previously [9]. UV–Vis spectra show the transition of coproheme (no hydrogen peroxide, orange) to heme *b* (three times excess of hydrogen peroxide, blue). The highest relative MMD concentration was identified at 2 eqiv. H_2_O_2_ (pink). Grey lines indicate equilibrium spectra obtained at H_2_O_2_ equivalents other than 2 or 3 (0.5, 1, 2.5). The inset shows the distribution of porphyrin species from the samples of the UV–Vis titration obtained by mass spectrometry.

*Cd*ChdC wild-type was reconstituted with coproheme and titrated with hydrogen peroxide to estimate the best oxidant concentration to obtain the maximum amount of MMD during reaction. Figure 3 shows that from the titration of *Cd*ChdC wild-type with hydrogen peroxide, the highest amount of MMD is obtained at a two-fold excess of the oxidant. In the production process, the reaction was stopped by the addition of cyanide at this hydrogen peroxide concentration and all porphyrins were extracted in order to be further purified by reversed-phase chromatography using an HPLC system coupled to a photo-diode array detector.

**Figure 4:**
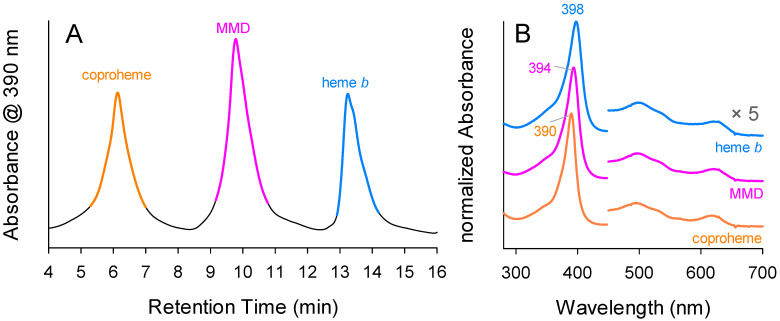
Purification of MMD. (**A**) Elution profile of porphyrins extracted by butanol from the enzymatic reaction. Coproheme peak is highlighted in orange, MMD in pink, and heme *b* in blue. (**B**) UV–Vis absorption spectra of eluates at the respective peak maxima. The coproheme spectrum was extracted at elution time of 6.1 min (orange line), MMD elution time of 9.8 min (pink line), and heme *b* of 13.2 min (blue line). The UV–Vis spectra were normalized and shifted for better visualization.

The obtained chromatograms show baseline separations of the three peaks corresponding to coproheme, MMD, and heme *b*, as expected from the activity measurements (Figure 4A). Spectra extracted from the peak maxima clearly identify the respective porphyrin species, with free coproheme having a Soret maximum at 390 nm, free MMD at 394 nm, and free heme *b* at 398 nm (Figure 4B). This agrees with the effect expected by porphyrin conjugation related to the present vinyl groups [30]. MMD-containing fractions were pooled after elusion and sent to mass spectrometry, which confirmed a purity of more than 95.1% of the enzymatically produced MMD, with 4.9% unreacted coproheme. The purity of MMD can be increased at the cost of the overall yield with respect to the initially used coproheme amount. The yield of this enzymatically produced MMD was approximately 40% of the initial coproheme amount.

### 3.2. Reactivity of MMD in CdChdC Wild-Type and the Y135A Variant

**Figure 5:**
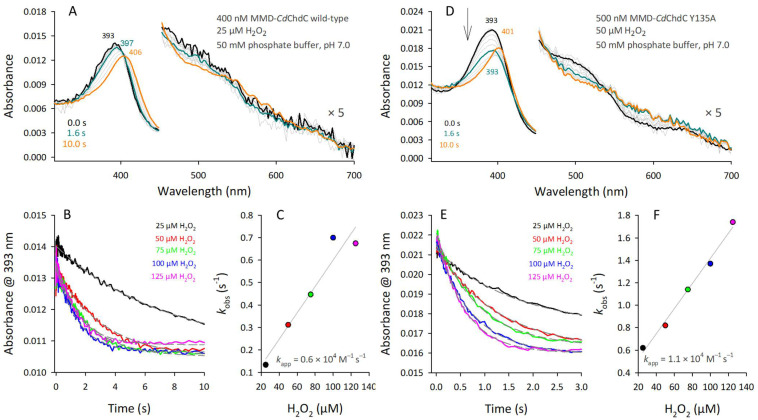
Compound I formation rate of MMD–C*d*ChdC wild-type and MMD–C*d*ChdC Y135A. (**A**) UV–Vis spectral transitions starting from MMD–C*d*ChdC wild-type (black line); transition spectrum after 1.6 s is shown in blue; final spectrum after 10 s is represented as orange line. (**B**) Time traces at 393 nm of MMD–C*d*ChdC wild-type at varying hydrogen peroxide concentrations; single exponential fits are shown as grey dashed lines. (**C**) Plot of *k*_obs_-values (s^−1^) versus the hydrogen peroxide concentration; linear regression allows the calculation of *k*_app_. Graphs (**D**–**F**) are assembled analogously to (**A**–**C**) and represent the reaction of hydrogen peroxide with MMD–C*d*ChdC Y135A variant.

*Cd*ChdC wild-type or the Y135A variant were reconstituted with the enzymatically produced MMD for pre-steady-state and steady-state reactions with various concentrations of H_2_O_2_. Starting from the three-propionate intermediate, the second dearboxylation step is monitored solely to see the formation of heme *b* in *Cd*ChdC wild-type. This is shown in Figure 5A–C. After a small, initially observed hypochromicity (decrease in extinction coefficient of the Soret band until 1.6 s), which allows us to estimate the Compound I formation rate, the formation of heme *b* is evident by the final spectrum obtained after 10 s (Figure 5A, orange line) with a Soret maximum at approximately 406 nm [9]. Estimation of *k*_app_, representing Compound I kinetics, yielded a rate of approximately 6 × 10^3^ M^−1^ s^−1^ (Figure 5B,C).

Analogous experiments on reactivity with H_2_O_2_ using MMD-reconstituted *Cd*ChdC Y135A allow us to follow Compound I formation even more precisely. Y135 is essential for catalysis and its elimination yields inactive protein which only reacts to Compound I (Figure 5D, blue line) and further decays to an oxidized coproheme *d* species by forming a lactone ring which has a Soret maximum at 401 nm, clearly distinct from the heme *b* spectrum (Figure 5D, orange line) [9,25,31]. Compound I is characterized by hyperchromicity of the Soret band with a maximum similar to the ferric resting state. The Compound I formation rate of MMD–C*d*ChdC Y135A can be followed at the decrease in the MMD–Soret maximum, and the initial phase was fitted single-exponentially (Figure 5E). The *k*_app_-rate was 1.1 × 10^4^ M^−1^ s^−1^ (Figure 5F), being in the range of, but still lower than, the Compound I formation rate of coproheme–*Cd*ChdC Y135A [9].

## 4. Discussion

Mechanistic data on the activity of coproheme decarboxylases, starting from the three-propionate intermediate monovinyl, monopropionate deuteroheme (MMD), are an important piece with which to fill the remaining gaps in the literature, which exist for the second part of this enzymatic multistep reaction. The actinobacterial *Cd*ChdC was shown to be a good model system to study mechanistic details because the obtained yields of purified recombinant proteins (wild-type and variants) allow us to employ biophysical and biochemical methods which are considered to be of high protein consumption [9,18].

In actinobacterial *Cd*ChdC, a distal Lewis base (H118), which is capable of activating the substrate hydrogen peroxide, was identified to yield Compound I. Oxidative Compound I formation of coproheme is the first step of the decarboxylation of propionate 2 (p2), which then results in the formation of a Compound I* (being characterized by a catalytic tyrosyl radical, Y135^●^). The second decarboxylation of p4 to yield the final product, heme *b*, was proposed to yield Compound I starting from MMD, which is now reported with experimental evidence in this work (Figure 5). The lack of the distal H118 slows down the Compound I formation rate significantly and therefore also the decarboxylation reaction [9] and the side reaction of coproheme *d* formation [19]. H118 is found on the loop which connects the N-terminal and the C-terminal ferredoxin-like folds of a ChdC subunit [24]. Such a residue has not been observed in firmicute ChdCs, which manifests in slower enzyme kinetic parameters [5]. This H118 not only acts as the distal base for hydrogen peroxide deprotonation and Compound I formation but is also of high importance for the structural integrity of the active site architecture. Alteration of this distal histidine to a bulky hydrophobic phenylalanine (H118F) results in the accumulation of MMD [13], which was exploited in this work in order to set up the production and purification necessary to enzymatically produce this three-propionate intermediate; in the end, we have utilized *Cd*ChdC wild-type due to its even higher yields, even though the formed heme *b* was a byproduct (it was discarded). Further, the H118F variant is unable to bind exogenous heme *b*, whereas the H118A loses specificity of orientation in heme *b* binding, showing the product also to be bound in the reversed configuration [18]. The exchange to a small alanine residue further affects the active site architecture and results in altered CO-binding interactions, mirroring the H-bonding network in the active site in the presence of a substrate or ligand [16]. This in-depth knowledge of the active site of *Cd*ChdC allowed us to successfully produce MMD enzymatically, which was more convenient for us than organic synthesis [6].

**Figure 6:**
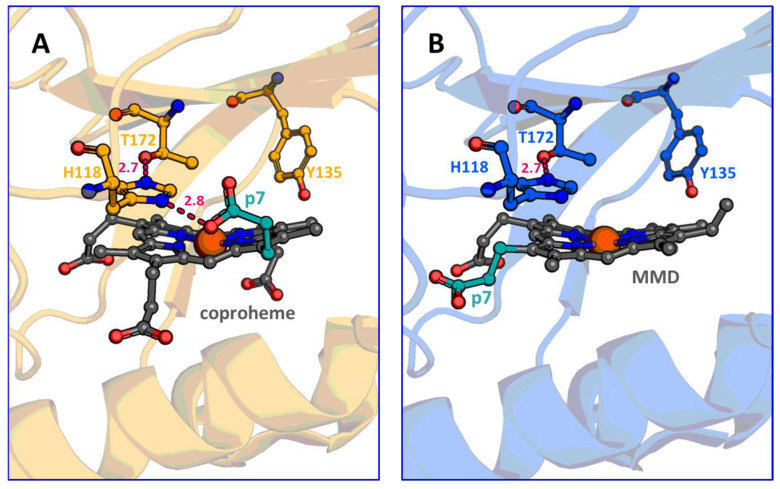
Active site of coproheme- and MMD–C*d*ChdC. (**A**) Active site of coproheme–*Cd*ChdC (pdb-code: 6XUC, yellow); distal active site carbon residues are displayed as yellow ball and stick models and coproheme as grey ball and stick models (p7 is shown in turquoise). (**B**) Active site of MMD–C*d*ChdC (pdb-code: 6XUB; blue); distal active site carbon residues are displayed as blue ball and stick models and coproheme as grey ball and stick models (p7 is shown in turquoise). All oxygen atoms are presented in red, nitrogen atoms in dark blue, and dashed lines for H-bonding interactions are shown in pink.

The MMD–ChdC complex was studied spectroscopically, albeit not during turnover, using the firmicute representative of *Staphylococcus aureus* [6]. Firmicute systems were also the first ones to be investigated mechanistically, delivering highly valuable insights on the first decarboxylation reaction starting from coproheme and allowing us to postulate that the second decarboxylation reaction starting from MMD is working analogously [7,8,10,11,14,15,20]. Here we report data in which, as expected, the second decarboxylation of p4 works identical to the first decarboxylation of p2. This is not surprising, given the fact that the catalytic tyrosine was shown to be essential for both decarboxylation reactions, and that the three-propionate intermediate MMD reorients in the active site prior to decarboxylation of p4 [9,11,13]. Nevertheless, a second Compound I formation could not be observed during the turnover of coproheme using the wild-type in the reaction with hydrogen peroxide. After the initial coproheme–Compound I formation, no further hypochromicity indicating a second, postulated Compound I formation, starting from MMD, was observed; only a steady-state shift towards the spectrum of the product, heme *b*, occurs and, clearly, no further pre-steady state transition can be followed [9]. This is now rationalized, as the Compound I formation rate starting from MMD, measured using the Y135A variant, is slightly below (1.1 × 10^4^ M^−1^ s^−1^, Figure 4) the one from coproheme–*Cd*ChdC Y135A (1.5 × 10^4^ M^−1^ s^−1^) [9]. The same hierarchy is observed when *Cd*ChdC wild-type is investigated. This can be reasoned by the fact that H118 is H-bonded to propionate on position 7 in coproheme–*Cd*ChdC in contrast to MMD–C*d*ChdC where H118 is not, due to the different orientation of MMD, within the active site [9,13] (Figure 6). Therefore, in coproheme–*Cd*ChdC, H118 is slightly more polarized than in MMD–C*d*ChdC, making it more efficient according to the Poulos–Kraut mechanism of Compound I formation [32]. Still, it has to be noted that the rate constant of Compound I formation is about three orders of magnitude lower than in the originally described “Push–Pull” mechanism for the heterolytic cleavage of hydrogen peroxide facilitated by the His–Arg pair in cytochrome *c* peroxidase [32,33,34].

Using the wild-type makes the comparison a bit more difficult, as coproheme undergoes two decarboxylation reactions compared to one when MMD is used as a starting substrate to form heme *b*. The Y135A variant, being unable to form Compound I*, stops after Compound I formation and only the side reaction of coproheme *d* formation (i.e., the oxidized coproheme lactone formation) is responsible of the final spectral shift towards 401 nm [25].

## 5. Conclusions

**Scheme 1:**
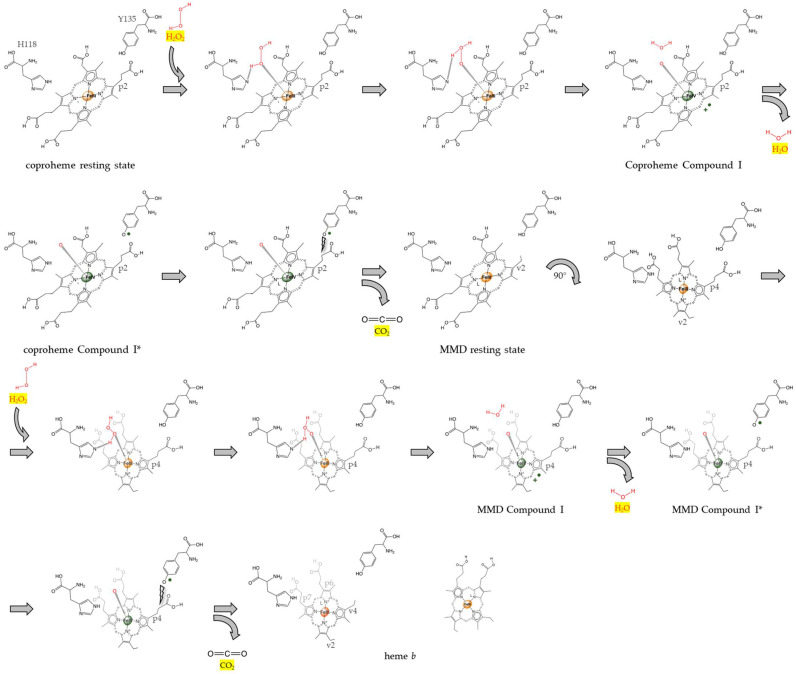
Reaction mechanism of coproheme decarboxylase as proposed by Michlits et al., 2020 [9], and explained in the discussion.

In this work, we were able to study the hydrogen peroxide-dependent reaction of the transient intermediate, MMD, in *Cd*ChdC wild-type and the Y135A variant under controlled conditions. The obtained results prove that the second decarboxylation reaction, starting from the transiently formed MMD intermediate, works in the same manner as the first one, which starts from the physiological porphyrin substrate, coproheme. The experimental evidence presented in this work proves the so-far-uninvestigated reactions of MMD–C*d*ChdC with hydrogen peroxide—that is, the initiation of the second decarboxylation step. To be more specific, it proves the previously proposed mode of catalytic action by showing the Compound I formation of MMD–C*d*ChdC upon reaction with an oxidant (e.g., hydrogen peroxide). In the work of Michlits et al. in 2020, the re-orientation of MMD was first shown by X-ray crystallography, which led us to believe that the second decarboxylation reaction works analogously to the first one [9]—a belief which is now further supported by the findings presented here. The overall reaction can be explained as follows: the reaction cycle is initiated by the oxidation of the ferric resting state of coproheme *Cd*ChdC upon reaction with hydrogen peroxide to form Compound I (oxoiron(IV) porphyryl radical) and water; histidine 118 accepts a proton and acts as a distal base that promotes heterolytic cleavage of H_2_O_2_; compound I is further instantaneously converted to Compound I* (oxoiron(IV) Y135^●^) by internal electron transfer; the neutral tyrosine radical performs a nucleophilic attack on the β carbon of propionate at position 2 (p2), consequently initiating its decarboxylation and formation of vinyl (v2); the resulting monovinyl monopropionate deuteroheme (MMD) undergoes a rotation of approximately 90°, which is important to move p4 in the position in ultimate proximity to Y135; the second half of the reaction cycle, which has been evidenced in this work, starts with the oxidation of the ferric resting state of MMD–C*d*ChdC by hydrogen peroxide to MMD–Compound I, which is immediately converted to MMD-Compound I*, which attacks p4, thereby initiating its decarboxylation and formation of vinyl (v4); the final product, heme *b*, is released and delivered to proteins. This reaction cycle is already visualized in previous works [9,24] and summarized in Scheme 1.

This study was enabled by the optimization of MMD production, using an enzymatic workflow and an easy-to-use and cheap protocol that once again impressively demonstrates how enzymatic reactions can be used to carry out complex organic reactions under environmentally friendly conditions.

## Data Availability

Not applicable.
